# Previous antibiotic exposure and antibiotic resistance of commensal *Staphylococcus aureus* in Spanish primary care

**DOI:** 10.1080/13814788.2018.1444748

**Published:** 2018-03-15

**Authors:** Albert Boada, Mariona Pons-Vigués, Jordi Real, Elisabet Grezner, Bonventura Bolíbar, Carl Llor

**Affiliations:** a Equip d’Atenció Primària Guinardó, Gerència d’Àmbit d’Atenció Primària Barcelona Ciutat, Institut Català de la Salut, Barcelona, Spain;; b Institut Universitari d’Investigació en Atenció Primària Jordi Gol (IDIAP Jordi Gol), Barcelona, Spain;; c Universitat Autònoma de Barcelona (UAB), Bellaterra (Cerdanyola del Vallès), Spain;; d Universitat de Girona, Girona, Catalunya, Spain;; e Centro de Investigacion Biomedica en Red de Diabetes y Enfermedades Metabolicas Asociadas, Barcelona, Spain;; f Universitat Internacional de Catalunya, Epidemiologia i Salut Pública, Barcelona, Catalunya, Spain;; g Institut Catala De La Salut, Laboratori Clínic L’Hospitalet, Barcelona, Catalunya, Spain

**Keywords:** Antibiotics, *Staphylococcus aureus*, carriage, resistance, primary health care

## Abstract

**Introduction:** Commensal flora of healthy people is becoming an important reservoir of resistant bacteria.

**Objectives:** To evaluate the relationship of previous antibiotic-dispensed and resistance pattern of strains of *Staphylococcus aureus* in primary care patients.

**Methods:** A cross-sectional study was carried out in seven primary care centres in Catalonia, Spain, from October 2010 to May 2011, as part of the APRES (The appropriateness of prescribing antibiotics in primary care in Europe concerning antibiotic resistance) study. Outpatients aged 4 or more who did not present an infectious disease and had not taken antibiotic or had not been hospitalised in the previous 3 months were invited to participate. Nasal swabs were collected for *S. aureus* culture, and antimicrobial susceptibility testing was carried out. Antibiotics dispensed boxes in the previous 4 years were extracted from Information System for Research in Primary Care.

**Results:** A total of 4,001 nasal swabs were collected, and 3,969 were tested for identification, 765 *S. aureus* were tested for resistance. Resistance rates to penicillin, azithromycin and methicillin were 87.1%, 11.6% and 1.3%, respectively, and a total of 10 MRSA strains were isolated (1.3%). Penicillin-resistant staphylococci were statistically significantly associated with the previous number of packages of penicillin dispensed (OR, 1.18; 95% CI, 1.04–1.35).

**Conclusion:** Although no causal inference is possible, an association was observed between previous antibiotic dispensation and isolation of resistant organisms in community-dwelling individuals, mainly between packages of penicillin and penicillin-resistant staphylococci.

KEY MESSAGES

- One-fifth of the individuals are carriers of *Staphylococcus aureus*, with the percentage being higher among children
- Methicillin-resistant *Staphylococcus aureus* is still uncommon in primary care
- A trend towards a relationship between the previous use of antibiotics and the isolation of resistant microorganisms in otherwise healthy carriers was observed in this study

## Introduction

Approximately 85% of antibiotics in Spain are prescribed to outpatients, but information available on the antibiotic resistance profile is nearly exclusively based on specimens from hospitalised patients, mainly those with severe and invasive infectious diseases [1]. It is well established that the prevalence of resistance to antibiotics greatly depends on their use since increased consumption of these drugs may not only produce higher resistance at the individual patient level but may also spur greater resistance at the community level, which can harm individual patients. The relationship between antibiotic consumption and the emergence of resistant pathogenic germs is well established [1,2], but evidence about previous antibiotic use regarding the isolation of resistant commensal germs in otherwise healthy individuals is scarce.

*Staphylococcus aureus* is a commensal organism that is widely distributed in nature. Approximately 20%–30% of healthy people carry this organism, mostly in the nose [3]. Carriage of *S. aureus* rarely causes disease in healthy individuals, but it is associated with an increased risk for the emergence of infections in various populations. Individuals in the community mostly become infected with their own carriage strain [4]. Since the introduction of antibiotics, *S. aureus* has quickly become resistant to many relevant antibiotics such as β-lactams and macrolides [4,5]. Antibiotic-resistant strains, such as methicillin-resistant *S. aureus* (MRSA), have emerged as a significant threat in both the hospital and community settings [6]. Several studies in healthy individuals in the general population and in children or specific groups have shown resistance to *S. aureus* [7–12]. Therefore, studies on the probable association between consumption of antibiotics by the individual and resistance to *S. aureus* in the general population are needed.

The APRES (The appropriateness of prescribing antibiotics in primary care in Europe concerning antibiotic resistance) project aimed at providing information and recommendations on the appropriateness of prescribing antibiotics in primary health care in nine European countries and was funded by the European Commission [12]. Several papers on the prevalence of *S. aureus* and pneumococcus carriage, and the association with demographic variables and antibiotic resistance, have been published so far [10–15]. However, information about previous individual antibiotic exposure was only available in Spain.

Therefore, we evaluated the association between previous antibiotic reimbursement data with antibiotic resistance profiles in healthy individuals with nasal carriage of staphylococci in primary care in Catalonia (Spain).

## Methods

We conducted a multi-centre cross-sectional study. The methodology of the APRES study has been described elsewhere [10,16]. Briefly, we studied the relationship of antibiotic prescription reimbursement data with the results of the samples collected during the APRES study of participants in 27 practices (18 GPs, eight paediatricians and one primary care nurse) from seven different primary healthcare centres in Catalonia in 2010–2011. Clinicians recruited patients aged 4 or more years who did not present any sign of infectious disease and had not taken any antibiotic or had not been admitted to any health centre in the previous 3 months. To obtain samples during the study period from October 2010 to May 2011, individuals were selected at a rate of about 12 patients per week in family medicine or nurse consultations and three patients per week in paediatric care consultations from Monday to Thursday. We did not register patients who declined to participate.

Nasal swabs were collected from all patients according to an established protocol [16,17]. Samples were sent to two different laboratories of the study area: Laboratori Clínic l’Hospitalet and Laboratori Clínic Bon Pastor. All the *S. aureus* isolated were tested in a central laboratory (Maastricht University Medical Centre, the Netherlands) for susceptibility to 12 antibiotics assumed to represent a range of commonly used antibiotic classes. The procedure included standardised microdilution tests and classification afterwards (resistant versus susceptible) was based on the breakpoints of the minimum inhibitory concentrations (MIC) of *S. aureus* defined in the 2017 guidelines of the European Committee on Antimicrobial Susceptibility Testing (EUCAST), published by the European Society of Clinical Microbiology and Infectious Diseases, the European Centre for Disease Prevention and Control and the European national breakpoints committees [17] (Table 1). Breakpoints for *S. aureus* have not changed since 2011. The genotypic structure of the isolated MRSA strains was established with the spa typing method [10].

**Table 1.** EUCAST definitions of clinical breakpoints issued by the 2017 European Committee on Antimicrobial Susceptibility Testing (EUCAST) and the number of isolates studied.

	EUCAST breakpoints*		Resistance of the samples collected from the carriers	
Antibiotic	S ≤ mg/L	R > Mg/L	S *N* (%)	R *N* (%)
Penicillin	0.125	0.125	99 (12.9)	666 (87.1)
Oxacillin	0.25	2	755 (98.7)	10 (1.3)
Erythromycin	1	2	679 (88.8)	86 (11.2)
Azithromycin	1	2	676 (88.4)	89 (11.6)
Clindamycin	0.25	0.5	691 (90.3)	74 (9.7)
Ciprofloxacin^1^	1	1	747 (97.6)	18 (2.4)
Gentamicin	1	1	757 (99.0)	8 (1.0)
Tetracycline	1	2	751 (98.2)	14 (1.8)
Vancomycin	2	2	765 (100)	0 (-)
Linezolid	4	4	765 (100)	0 (-)
Daptomycin	1	1	765 (100)	0 (-)

S = susceptible; R = resistant.

* The figures correspond to the minimal inhibitory concentrations (MIC) in mg/L above, which germs are considered as resistant.

1 Breakpoints are based on high dose therapy (750 mg ×2).

The boxes of antibiotics reimbursed in the previous 4 years were extracted from the Information System for Research in Primary Care (SIDIAP Database), which contains the computerized primary care medical records of approximately 80% of the Catalan population and data on dispensation of publically financed medication. Despite the availability of the daily defined dose (DDD) of the different antibacterial agents in the same 4-year period, we preferred the use of boxes as suggested by the European Centre for Disease Prevention and Control because of the difficulty of using DDDs for children, invalid for paediatric formulations [18].

### Statistical analysis

Descriptive statistics were used to analyse the individuals’ characteristics and the antibiotic-resistance patterns of the staphylococci isolated. The prevalence of the resistance rates and the number of boxes of the different antibiotics prescribed in the previous 4 years were calculated. To evaluate the association of the previous number of boxes dispensed and the staphylococcal resistance patterns, a multivariate logistic regression analysis model was constructed with resistance of isolated *S. aureus* strain as the dependent variable and considering age, sex and number of boxes of the different antibiotics prescribed (according to the Anatomical Therapeutic Chemical Classification System) in the prior 4 years as independent variables. Adjusted odds ratios (ORs) with their 95% confidence interval (95% CI) were estimated. Antibiotics evaluated were penicillin, oxacillin, erythromycin, azithromycin, clindamycin, ciprofloxacin, gentamicin, tetracycline, vancomycin, linezolid and daptomycin. Differences were considered to be significant with *P* < 0.05.

The study was approved by the Research Ethics Committee IDIAP Jordi Gol Clinic (P10/55), and each participant voluntarily signed the informed consent. In the case of minors, the father or mother or legal guardian was the one who signed it.

Anonymity guaranteed and confidentiality, as well as the protection of data.

## Results

### Demographic characteristics

A total of 4,001 nasal swabs were collected, and 3,969 were tested for identification. Finally, 776 (19.6%) were *S. aureus* positive, of which 765 samples (19.3%) (95% CI, 18.1–20.5) were tested for resistance. The mean age of evaluated carriers was 43.2 years (SD: 23.1). Carriage was higher in children less than 14 (136 cases; 35.6%) than in individuals aged 15–64 (459 cases; 19.4%) and people more than 65 years old (170 positive cases; 14%). Among the 765 evaluated subjects colonised with *S. aureus*, 370 (48.2%) were male.

### Antimicrobial susceptibility

Based on the antimicrobial susceptibility reports among the 765 evaluated *S. aureus* isolates, 10 (1.3%; 95% CI, 0.7%–2.4%) were classified as MRSA. The highest resistance rates were observed to penicillin, followed by azithromycin, erythromycin and clindamycin, with resistance percentages of 87.1%, 11.6%, 11.2%, and 9.7%, respectively. A total of 1.3% strains were resistant to oxacillin (Table 1); 83 strains did not exhibit any resistance to the antibiotics tested (10.8%).

### Association with previous antibiotic exposure

Of the 765 individuals harbouring *S. aureus* strains, 208 were found not to have taken antibiotics in the previous 4 years (27.1%). Of the subjects, 18.1% had taken one box, 13.0% two boxes, 11.5% three boxes of antibiotics, and 30.3% more than three boxes. The mean number of boxes of antibiotics consumed in the previous 4 years was 3.3 (SD: 5.2 and range 0–66), with penicillin being the most frequent antibiotic taken (mean of 1.5 packages; SD: 2.3), followed by ophthalmological presentations including antibacterial drugs (mean of 0.5 containers; SD: 2.9) and topical antibiotics (0.3 boxes; SD: 0.8).

Although not statistically significant, a trend was shown in which patients who had more than one antibiotic dispensed in the previous 4 years tended to harbour more staphylococcal strains resistant to any antibiotic compared to those without using antibiotics (OR, 1.07; 95% CI, 0.99–1.16; *P* = 0.55). Isolation of penicillin-resistant staphylococci was significantly associated with the previous number of packages of penicillin (OR, 1.18; 95% CI, 1.04–1.35) but not with other antibiotics packages. No association was observed between the number of boxes among individuals harbouring *S. aureus* resistant to oxacillin, clindamycin or azithromycin. However, we did observe a statistically significant association between harbouring MRSA and the previous prescription of any antibiotics (OR, 1.6; 95% CI, 1.16–2.20) (Table 2).

**Table 2.** Association of the previous number of antibiotics boxes dispensed and the staphylococcal resistance. A multivariate logistic regression analysis model.

Variable	95% CI	OR	*P* value
Staphylococcus aureus resistance			
Age (years)	0.98–1.00	0.99	0.24
Sex (women)	0.45–1.14	0.72	0.16
Any antibiotics^a^	0.99–1.16	1.07	0.55
Penicillin-resistant Staphylococcus aureus			
Age (years)	0.99–1.01	1.00	0.76
Sex (women)	0.46–1.01	0.70	0.11
Penicillin packages (number)	1.04–1.35	1.18	0.01
Methicillin-resistant Staphylococcus aureus (MRSA)			
Age (years)	0.99–1.05	1.02	0.22
Sex (women)	0.15–2.43	0.61	0.48
Any antibiotics^a^	1.16–2.20	1.60	<0.01

^a^Have taken one or more antibiotic boxes.

## Discussion

### Main findings

We found an average *S. aureus* nasal colonization rate of 19.3%. Except for the known high resistance against penicillin, the highest recorded resistance rate was found for azithromycin, and a total of 10 MRSA strains were isolated (1.3%). Despite not observing statistically significant differences, a trend towards a relationship between the dispensation of antibiotics in the previous 4 years and the isolation of resistant microorganisms in otherwise healthy carriers was observed in this study. However, a significant association was found between previous number of packages of penicillin dispensed and penicillin-resistant strains of *S. aureus*.

### Limitations and strengths

There are a number of limitations to this study. Although the most important limitation of this study was that the fieldwork was conducted in 2010 and 2011, we used the current resistance breakpoints published in 2017. We do not think the results would have been different if the samples had been collected more recently, since the consumption of antibiotics in our country has been stable over the last years and the main aim of the present study was to determine a possible relationship between previous antibiotic exposure and the prevalence of relevant resistant strains in the noses of healthy individuals. On the other hand, since we carried out a cross-sectional study and compared the results of the resistance patterns of the staphylococci harboured with previous antibiotic exposure, we cannot infer any causality effect between antibiotic exposure and resistance. However, we were able to compare the number of boxes of antibiotics previously dispensed and the resistance patterns of commensal staphylococci in otherwise healthy individuals.

Another limitation of the study is the fact that we considered antibiotics dispensed in the previous 4 years. Prescription of antibiotics based on reimbursement data can mimic antibiotic consumption in most European countries, but is not true in southern European countries. A landmark paper published in 2007 with the use of sales data in 2002–2005 showed a difference between prescription and consumption of up to 30% in Spain, mainly due to the over-the-counter sale of antibiotics without a medical prescription and antibiotics prescribed by the private sector [19]. A higher antibiotic consumption would very likely have been associated with greater resistance rates to antibiotics.

Another weakness is that this study monitors colonisation at a single time-point, although colonisation is known to be variable over time and it should be differentiated between persistent and intermitted carriers [20]. Only nasal swab samples were used to determine *S. aureus* colonization status, as the study protocol did not include swabbing of extranasal body sites. While the prevalence might be underestimated by using nasal swabs, we assume our final sample of *S. aureus* to be representative of all carriage. We also assume that antimicrobial resistance patterns in the commensal flora of the nose are comparable with isolates of pathogenic staphylococci. However, for purposes of empirical treatment, data on resistance of pathogenic strains would also be required.

The greatest strength of this work is that it is a study in primary care patients. We are therefore able to extrapolate these results to what we observe in primary care.

### Comparison with other studies

#### S. aureus and MRSA carriers

We found an average *S. aureus* nasal colonization rate of 19.3%, which is comparable to previously described colonization rates in the general population in Europe and the United States [21,22]. The low prevalence of MRSA isolates, 10 (1.3%) was also found to be consistent with studies from Europe. We observed a positive association between previous dispensation of any antibiotics and isolation of MRSA, but caution is needed as MRSA was only isolated in 1.3% of the individuals in our study. This is consistent with previous hospital studies where antibiotic surgical prophylaxis increases nasal carriage of antibiotic-resistant staphylococci [23]. Evidence seems to indicate that the endogenous microflora of the patient may be critical since clinical studies have found that *S. aureus* skin colonisation increases the risk of a subsequent infection by three-fold, and up to 80% of cases of staphylococcal bacteraemia are caused by strains identical to those in the patient’s nasal cavity [24]. Furthermore, patient colonisation with *S. aureus* is associated with a 2–9-fold increased risk of infection [25].

#### Antibiotic consumption and antibiotic-resistant association

The association between antibiotic consumption and antibiotic-resistant organisms has been widely observed, mainly for other respiratory tract pathogens. For instance, Granizo et al. observed a clear association between previous use of macrolides and β-lactam with erythromycin-resistant pneumococci in Spain [26]. Also, Malotra et al. note that azithromycin and erythromycin use increases resistance of streptococci in healthy carriers [27]. We already knew of the relationship between antibiotic prescribing and bacterial resistance in primary care when antibiotics were prescribed for respiratory or urinary infection [2]. Other studies have also shown an association between previous consumption of antibiotics and *S. aureus* resistance. For example, in a recent randomised controlled trial, Australian children diagnosed with bronchiectasis assigned to intermittent azithromycin consumption showed higher macrolide-resistant *S. aureus* carriage than those assigned to placebo [28].

The commensal flora of community-dwelling persons is, therefore, becoming an important reservoir of resistant bacteria. Fighting antibiotic resistance starts with the restricted use of antibiotics, leading to less selection pressure on the bacterial flora circulating in the population, but also on the commensal flora in an individual. Knowledge of the local resistance rates of the most common organisms to antibiotics is crucial for better prescribing of antibiotics. In a recent paper from the APRES project, van Bijnen et al. observed that many guidelines for skin infections do not contain data on local resistance [29]. GPs should be informed of the resistance profiles of the most frequent organisms causing infectious disease for more prudent use of antibiotics.

## Conclusion

A statistically significant relationship was observed between the number of boxes of penicillin dispensed and staphylococcal resistance towards penicillin. Although a causal inference between previous exposure to antibiotics and acquisition of resistant organisms cannot be drawn in this study, we also observed a trend for a higher dispensation of boxes of antibiotics among individuals with resistant organisms compared to those with susceptible staphylococci.
